# Genomic Characterization of NDM-1 Producer *Providencia stuartii* Isolated in Russia

**DOI:** 10.3390/antibiotics14121238

**Published:** 2025-12-08

**Authors:** Valeria V. Shapovalova, Vladimir A. Ageevets, Irina V. Ageevets, Alisa A. Avdeeva, Ofeliia S. Sulian, Alina D. Matsvay, Yuliya A. Savochkina, Ekaterina N. Belyakova, German A. Shipulin, Sergey V. Sidorenko

**Affiliations:** 1 Federal State Budgetary Institution «Centre for Strategic Planning and Management of Biomedical Health Risks» of the Federal Medical Biological Agency, 119121 Moscow, Russia; 2 Federal Research and Clinical Center for Infectious Diseases, 197022 Saint Petersburg, Russia; 3 Department of Medical Microbiology, North-Western State Medical University Named After I.I. Mechnikov, 191015 Saint Petersburg, Russia

**Keywords:** *Enterobacterales*, antibiotic resistance, *bla_*NDM*_*, *Providencia stuartii*, IncC plasmid

## Abstract

**Background/Objectives:** *Providencia stuartii* is intrinsically resistant to several antibiotic classes, and acquisition of *bla*_NDM_ further restricts treatment options. This study aimed to characterize NDM-producing *P. stuartii* isolates from a small hospital cluster in Russia and to place them within the global genomic context. **Methods:** Four isolates recovered between June and July 2023 from a single hospital were analyzed using Illumina and Oxford Nanopore sequencing to assess genetic relatedness and plasmid content. **Results:** The isolates showed identical extensively drug-resistant profiles and were closely related genomically. All carried nearly identical IncC plasmids harboring multiple antimicrobial resistance genes, including *bla*_NDM_. Comparative analysis indicated that these genomes clustered with recent European isolates but differed in the *bla*_NDM_ allele and its genomic location. Highly similar IncC plasmids were also found in our previous *Klebsiella pneumoniae* dataset, demonstrating that this plasmid backbone occurs in multiple bacterial species in the region. **Conclusions:** The study highlights the role of IncC plasmids in carbapenemase dissemination and underscores the value of genomic surveillance integrating chromosomal and plasmid analyses to track extensively drug-resistant pathogens.

## 1. Introduction

*Providencia stuartii*, an opportunistic pathogen within the Morganellaceae family, is associated with hospital-acquired infections, such as urinary tract infections, wound infections, and bacteremia, particularly in immunocompromised patients or those with indwelling devices [1]. It is intrinsically resistant to polymyxins, tigecycline, and several β-lactams due to chromosomal AmpC β-lactamases, which further complicates treatment [2]. The acquisition of carbapenemase genes therefore dramatically reduces the remaining therapeutic options and increases the risk of treatment failure [3].

Among the mobile genetic elements involved in carbapenemase dissemination, IncC plasmids are of particular concern because they readily transfer between diverse Gram-negative hosts and are frequently associated with *bla*_NDM_ and other clinically significant resistance determinants [4,5,6]. Their importance in *Providencia* has recently been underscored by a genus-wide genomic analysis showing that plasmid clusters related to IncC conjugative backbones carried more than one-third of all antibiotic resistance genes and the majority of carbapenemase genes across the genus [7]. Collectively, these findings suggest that IncC plasmids may play a central role in shaping the emerging resistome of *Providencia* spp.

Carbapenemase-producing *Providencia* has historically been rarely detected in global surveillance datasets. In the ATLAS program (39,368 Enterobacterales collected from 55 countries in 2018–2019), *Providencia* spp. accounted for 959 isolates (2.4%), including only 18 metallo-β-lactamase-producing strains [8]. Despite this low prevalence, reports of *P. stuartii* carrying carbapenemases—including NDM, VIM, OXA-48, IMP, and KPC—have increased across multiple countries in recent years [2,9]. This trend has been especially evident in Europe, with hospital-associated cases reported in Denmark [10], the Netherlands [11], France [12], Italy [13], Bulgaria [14], Romania [15], etc. A recent multinational investigation further demonstrated the cross-border dissemination of extensively drug-resistant NDM-producing *P. stuartii* across Europe [16]. In parallel, an ECDC risk assessment emphasised that current evidence remains limited and that additional genomic studies of carbapenem-resistant *P. stuartii* are urgently needed in Eastern Europe and neighbouring regions [17,18].

Understanding whether these high-risk *P. stuartii* lineages and their associated IncC plasmids have expanded into previously unsampled geographic regions is essential for accurate assessments of their dissemination. Detailed genomic characterization—including complete chromosome and plasmid reconstruction—remains limited in many regions and is essential for tracing the dissemination of these lineages, identifying conserved modules associated with *bla*_NDM_ carriage, and contextualizing local isolates within the global population structure.

Herein, we report a high-resolution genomic investigation of NDM-producing *P. stuartii* identified in Russia, representing a small cluster of clinically related cases from an intensive care unit. Using hybrid long- and short-read assemblies, we resolved complete chromosomes and plasmids, characterized the full resistome, and examined the structural diversity of their IncC backbones. By placing the isolates in a global phylogenetic framework, we show how these strains relate to internationally circulating *P. stuartii* lineages and clarify the contribution of IncC plasmids to their dissemination. This study fills an important regional knowledge gap within the broader epidemiological context on the dissemination of NDM-carrying IncC plasmids within *Providencia*.

## 2. Results

### 2.1. Case Overview

Between 13 June 2023 and 27 July 2023, four cases of infection sustained by NDM-producing *P. stuartii* were identified at one hospital in Russia. Table 1 outlines the summary characteristics of the patients.

All four patients were hospitalized in the same intensive care unit (ICU) of the hospital. Although their exact hospitalization routes and precise overlap periods were not available, the occurrence of all cases within the same ICU over a six-week period is consistent with possible ICU-associated transmission. Three isolates were from wounds, and one was from a central venous catheter. Three patients were female, aged 56–82 years (median: 60), and one patient was a 33-year-old male.

Antimicrobial susceptibility testing (AST) revealed that the strains were susceptible to aztreonam/avibactam and trimethoprim–sulfamethoxazole (Supplementary Table S1). The isolates were non-susceptible (I, “susceptible, increased exposure”, or R, “resistant”) to cefepime, ceftazidime, ceftazidime/avibactam, aztreonam, imipenem, meropenem, gentamicin, amikacin, and ciprofloxacin. PCR amplification for detecting *bla*_NDM_ was positive for all isolates.

### 2.2. Genome Features, IncC Plasmid Variation, and Resistome Profiling

**Figure 1:**
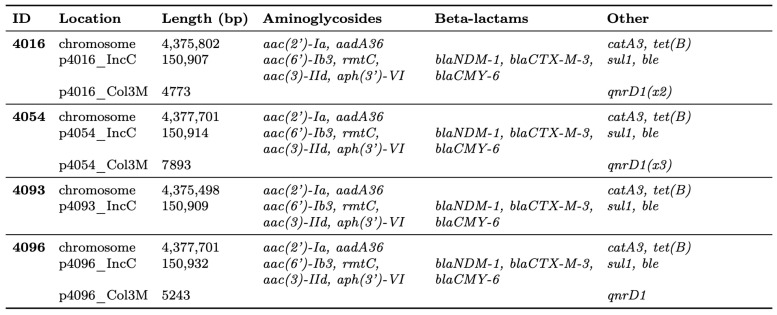
Genomic features of the four complete *P. stuartii* genomes.

Hybrid assembly yielded complete chromosomes and plasmids for all four isolates (Figure 1).

**Figure 2:**
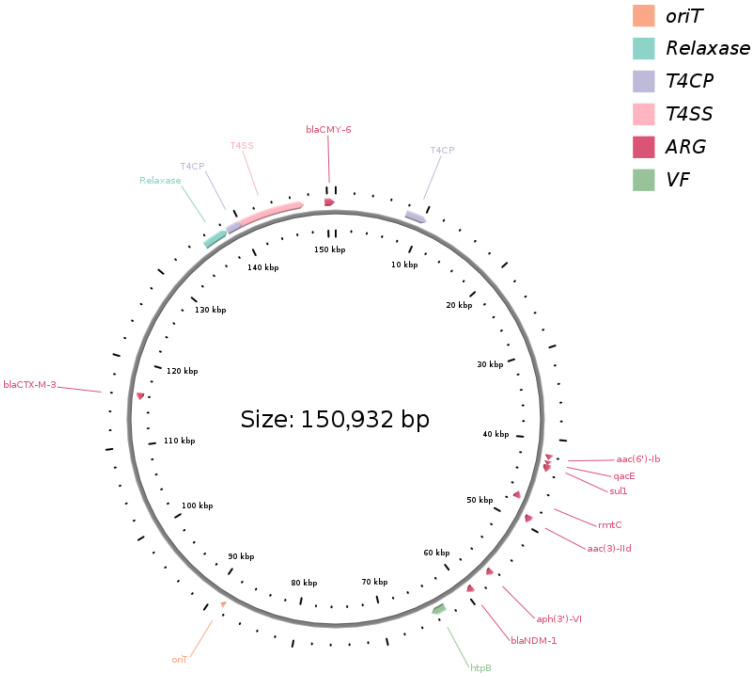
Circular representation of plasmid p4096_IncC (150,932 bp), shown as representative of four nearly identical plasmids. Annotation was performed with oriTfinder. Colored blocks indicate oriT (orange), relaxase (turquoise), type IV coupling proteins (T4CP, violet), type IV secretion system components (T4SS, pink), antimicrobial resistance genes (ARG, red), and virulence factors (VF, green). Key resistance determinants include *bla_*NDM-1*_*, *bla_*CMY-6*_*, and *bla_*CTX-M-3*_* and multiple aminoglycoside resistance genes.

Whole-genome sequencing confirmed that all isolates belonged to *Providencia stuartii*. Pairwise SNP distances ranged from 1 to 6 across 4190 core genes, consistent with very close genomic relatedness. Each genome carried a ~150 kb IncC plasmid with nearly 100% sequence identity and coverage, predicted to be conjugative (Figure 2).

Using plasmid p4096_IncC as the reference, whole-plasmid comparisons of p4016_IncC, p4054_IncC, and p4093_IncC revealed two variants shared by all three plasmids and one plasmid-specific change each. All three carried a 4 bp insertion at position 61,248 in the ampC–blc intergenic region, and a 16 bp deletion was observed at position 66,745 within a hypothetical protein, producing a frameshift. Unique to p4016_IncC was a 13 bp deletion at position 69,782 within *traC*, resulting in a frameshift. The *traC* gene encodes a component of the type IV secretion system required for conjugative transfer. Previous studies have shown that mutations in *traC* or *traD* can reduce conjugation efficiency [19]. Whether the deletion observed here affects conjugation remains unknown and was not assessed in this study. Unique to p4054_IncC was a 6 bp in-frame deletion at position 82,482 within a hypothetical protein, whereas p4093_IncC carried an 11 bp deletion at position 96,508 within a hypothetical protein, causing a frameshift.

All isolates exhibited an identical resistome, comprising 15 antibiotic resistance genes (ARGs) conferring multidrug resistance (MDR) to aminoglycosides, quinolones, β-lactams, phenicols, tetracyclines, sulfonamides, bleomycins, and disinfectants. The IncC plasmids carried most of the identified ARGs, including *bla*_NDM-1_, *bla*_CTX-M-3_, *bla*_CMY-6_, *rmtC*, *aac(6′)-Ib3*, *aac(3)-IId*, *aph(3′)-VI*, *qacEΔ1*, *sul1*, and *ble*. Chromosomally encoded resistance genes comprised *aac(2′)-Ia*, *aadA36*, *catA3*, and *tet(B)*.

Three isolates carried small Col3M-type plasmids that differed in size (4773 bp; 5243 bp; 7893 bp), but they exhibited nearly identical GC content (41.4–41.9%). All of them encoded qnrD-family quinolone resistance genes, with variations in copy number and structure. The plasmid from isolate 4096 contained a single *qnrD1* allele (100% identity), the plasmid from isolate 4016 carried two full-length *qnrD1* copies, and the largest plasmid (isolate 4054) encoded three qnrD-family genes, including two full-length *qnrD1* alleles and one truncated variant with an internal stop codon. No other resistance genes or mobilization-associated genes were detected.

### 2.3. Global Phylogenetic Context

**Figure 3:**
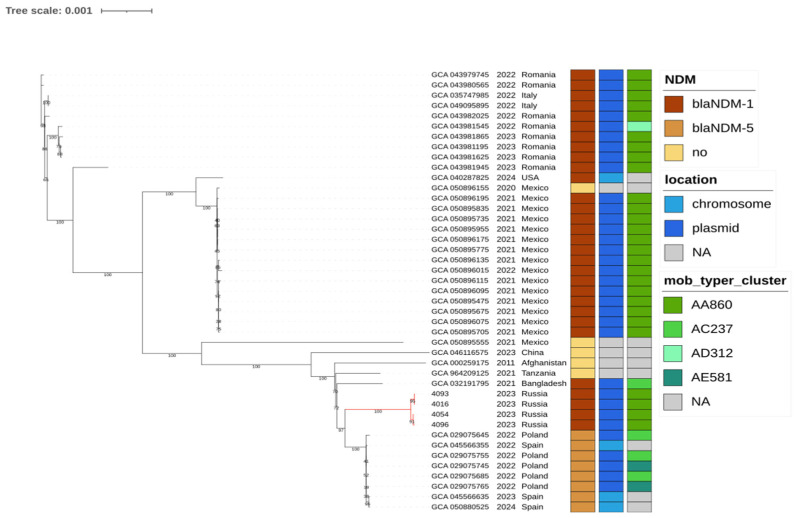
Core-genome phylogeny of *P. stuartii* FastBAPS cluster 1. The tree includes the 4 genomes from our study and 39 additional publicly available genomes. A maximum likelihood tree was constructed in IQ-TREE from the Panaroo-filtered core gene alignment with 1000 bootstrap replicates. The tree was midpoint-rooted, and branches corresponding to the outbreak clade are shown in red. The *mob_typer_cluster* legend indicates the primary cluster IDs assigned by the MOB-typer tool.

To contextualize the cluster isolates, a comparison was performed against all publicly available *P. stuartii* genomes (*n* = 494) from GenBank. Core-genome phylogeny combined with FastBAPS clustering revealed that the global population is structured into 16 clusters (Supplementary Figure S1A). The four cluster isolates were assigned to FastBAPS cluster 1, together with 39 additional genomes. Nearly half of the available genomes carried *bla*_NDM_ genes, with the majority located on plasmids (Supplementary Figure S1B). Plasmid typing using MOB-suite showed that the plasmids carrying *bla*_NDM_ from cluster 1 belonged to the same primary cluster (AA860) as plasmids found in multiple other clades, indicating that this plasmid lineage is widely disseminated across *P. stuartii* populations. To further resolve the relationships within this lineage, a separate core-genome phylogeny was reconstructed using only the genomes from FastBAPS cluster 1, which comprised the 4 genomes from our study and 39 additional publicly available genomes (Figure 3).

The additional genomes originated from human clinical samples collected between 2011 and 2024, but with only a single isolate before 2020, indicating that this lineage has expanded mainly in recent years. The isolates originated from ten countries, most frequently Mexico (*n* = 16), Romania (*n* = 8), and Poland (*n* = 5), with further representation from Spain (*n* = 3) and Italy (*n* = 2) and single genomes from Afghanistan, Bangladesh, the United States, China, and Tanzania. Reported infection types included bacteremia (*n* = 7), respiratory tract infections (*n* = 5), urinary tract infections (*n* = 2), and single cases from burn patients, colonization, and unspecified infections, while disease information was unavailable for 20 genomes. Within cluster 1, 38 of the 44 genomes (86%) carried *bla*_NDM_ genes. *bla*_NDM-1_ was the most prevalent variant (*n* = 30), predominantly plasmid-borne (29 plasmids and 1 chromosomal copy), followed by *bla*_NDM-5_ (*n* = 8, with 5 plasmid-borne and 3 chromosomally integrated). Only five genomes lacked *bla*_NDM_. These findings indicate that *bla*_NDM_ is nearly ubiquitous within this lineage, with plasmids serving as the principal vehicle for carbapenemase dissemination. The nearest clade comprised genomes from Poland and Spain, which differed from our isolates by 76–80 core-genome SNPs. According to the associated BioProject description, Polish genomes were obtained from carbapenemase-producing *P. stuartii* isolates recovered from patients of Ukrainian origin who were hospitalized in Poland in 2022. Together with the Spanish genomes, these isolates were recovered in 2022 and 2024, and all carried *bla*_NDM-5_, in contrast to the *bla*_NDM-1_ isolates reported here.

Taken together, the geographic distribution and recent sampling dates of these genomes indicate that FastBAPS cluster 1 represents a widely distributed lineage that has expanded mainly in recent years. The close phylogenetic relatedness between isolates from different countries suggests that this lineage may be associated with movement across healthcare systems, although specific transmission routes cannot be determined from available metadata. The high prevalence of plasmid-borne *bla_*NDM*_* further supports the key role of plasmids in maintaining carbapenem resistance within this group.

**Figure 4:**
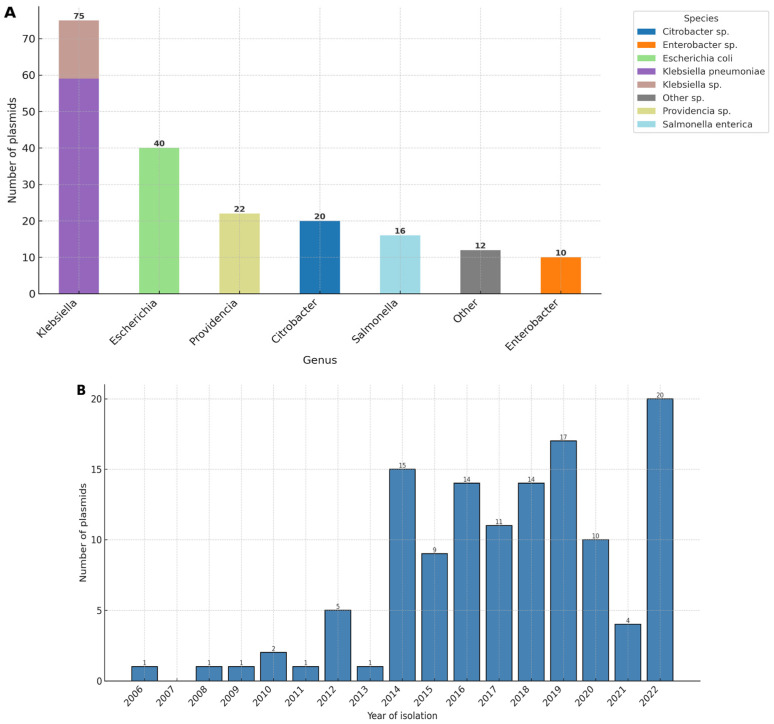
Distribution and temporal dynamics of plasmids highly similar to the newly sequenced IncC plasmids. (**A**) Host organisms from which the plasmids were isolated, as shown at the genus level with the most frequent species highlighted. (**B**) Temporal distribution of plasmid collection dates.

IncC plasmids carrying *bla*_NDM_ were compared against our custom plasmid database, which included all complete plasmids deposited in GenBank, in addition to complete plasmids from our previous studies on clinical *Klebsiella pneumoniae*. In total, 196 plasmids (Supplementary Table S2) were identified as highly similar to the newly sequenced plasmids (ANI > 99%, AF > 0.8). These plasmids originated from 12 Gram-negative genera, with the most common being *Klebsiella*, *Escherichia*, *Providencia*, and *Citrobacter* (Figure 4A).

Plasmids with available metadata were recovered from 28 different countries. The largest numbers were from China (*n* = 37) and the USA (*n* = 25), followed by India (*n* = 12), Bangladesh (*n* = 11), and Switzerland (*n* = 9). In addition, six plasmids were from Russia, all originating from *Klebsiella pneumoniae*: two from our previous work (isolated in 2022 from ST39 in one hospital) and four deposited by other groups (two isolated in 2019 and two in 2021). BLAST v.2.17.0 comparisons showed that the two plasmids from our previous dataset shared >99.9% nucleotide identity and >87% coverage with the newly sequenced IncC plasmids from *P. stuartii*, despite originating from a different hospital in another city (Supplementary Figure S2). Lower numbers were reported from a wide range of countries across Europe, Asia, Africa, Oceania, and North America. The temporal distribution shows the earliest isolates from the mid-2000s, followed by an expansion phase beginning in 2014 and peaking in 2022 (Figure 4B). The temporal distribution indicates that this plasmid type first appeared in the mid-2000s, followed by a period of limited detection until 2012. From 2014 onwards, the number of isolates increased rapidly, reflecting both wider dissemination and intensified genomic sequencing efforts. The highest number of plasmids was deposited in 2022, consistent with global surveillance initiatives and reported outbreaks during this period. Plasmids with available host information were overwhelmingly associated with humans (*n* = 93), while only a few were recovered from animals such as cattle (*n* = 3), swine (*n* = 1), poultry (*n* = 1), shrimp (*n* = 1), and a wild bird (*Milvus migrans*, *n* = 1). Analyses of isolation sources confirmed the predominance of clinical materials, including urine (*n* = 26), blood (*n* = 15), rectal swabs (*n* = 16), wound swabs (*n* = 9), stool (*n* = 7), and respiratory specimens (*n* = 8). A smaller subset originated from hospital and environmental reservoirs, such as hospital environments (*n* = 9), sewage/wastewater (*n* = 7), and water samples (*n* = 3). Only sporadic reports linked plasmids to food products (shrimp, beef burger, and turkey). Together, these data highlight that highly similar plasmids are primarily human- and hospital-associated, with limited detection in environmental, animal, or food-related contexts.

## 3. Discussion

*Providencia* species are opportunistic pathogens that primarily affect immunocompromised patients, and they are intrinsically resistant to several antibiotic classes, which limits treatment options. The emergence of carbapenem-resistant *P. stuartii* therefore represents a clinically significant challenge. Although *P. stuartii* remains relatively uncommon in hospital surveillance datasets, recent reports from Europe indicate an increasing number of cases involving this pathogen [16]. The 2024 WHO/ECDC antimicrobial resistance summary highlighted this trend and emphasized the need for the systematic surveillance of carbapenem-resistant *P. stuartii* across EU/EEA countries and neighbouring regions [18]. These observations provide important context for interpreting the findings of the present genomic investigation.

In this study, we genomically characterized NDM-producing *P. stuartii* from a region where such isolates had not been previously investigated. All four isolates belonged to a single lineage that also included recent genomes from Poland and Spain, confirming that this lineage is present in multiple European countries. Despite their close chromosomal relatedness, the isolates differed in both their *bla*_NDM_ allele and the genomic location of the gene. The observation that most genomes associated with this lineage were isolated after 2020 supports the recent emergence of a *P. stuartii* lineage enriched for *bla*_NDM_ carriage.

The IncC plasmids identified here were nearly identical and belonged to MOB-typer primary cluster AA860. This plasmid backbone appeared across multiple *P. stuartii* lineages in the global dataset, indicating that it is not restricted to a single genomic background. Prior reports have linked AA860-type IncC plasmids to multi-species hospital outbreaks and to carbapenemase dissemination [20]. Similar plasmids have been described in both *P. stuartii* and *K. pneumoniae* during an outbreak in Rome [13], and closely related IncC plasmids have also been detected in *K. pneumoniae* ST39 in our previously published work from a different Russian hospital [21]. Together, these observations support the recurrent appearance of this IncC backbone across different bacterial backgrounds and contexts.

Col3M plasmids carrying qnrD-family genes were found in three of the four isolates. Recent studies have identified Col3M as the most frequent plasmid type within the *Providencia* genus, with GC content closely matching that of the host, consistent with a long-standing association rather than recent acquisition. Variations in qnrD copy numbers across the Col3M plasmids observed here reflect structural plasticity within this plasmid type, and they may be shaped by local selective pressures or recombination events.

This study has several limitations. First, the analysis was based on only four isolates from a single hospital, limiting the epidemiological resolution and potentially underrepresenting the local diversity of carbapenem-resistant *P. stuartii*. Second, functional assays such as conjugation experiments were not conducted; thus, the biological impact of the observed *traC* frameshift and other plasmid variants cannot be determined. Third, comparative analyses relied on publicly available genomes and plasmids, which may contain sampling biases and uneven geographic representation.

Despite these limitations, the findings have potential relevance for infection control. The recurring detection of closely related IncC plasmid backbones in different bacterial backgrounds highlights the value of integrating plasmid-focused analyses into routine genomic surveillance. Such approaches may assist healthcare facilities in identifying high-risk resistance elements that move across species and settings, thereby informing targeted prevention and control strategies.

## 4. Materials and Methods

### 4.1. Bacterial Strains and Antimicrobial Susceptibility Testing

In June and July 2023, four carbapenem-resistant *P. stuartii* isolates were referred to our laboratory. The isolates were recovered from clinical samples as part of routine microbiological diagnostics. Species identification was performed using MALDI-TOF MS (Bruker Daltonics, Bremen, Germany). Antimicrobial susceptibility testing (AST) was performed once per isolate (no technical replicates), and MICs were interpreted according to EUCAST v15.0 breakpoints. The following antibiotics were tested: cefepime, ceftazidime, ceftazidime/avibactam, aztreonam, aztreonam/avibactam, imipenem, meropenem, biapenem, gentamicin, amikacin, tigecycline, ciprofloxacin, and trimethoprim-sulfamethoxazole. The detection of the blaNDM-1 gene was performed via PCR using previously described primers and reaction conditions [22]. Amplicons were confirmed via gel electrophoresis.

### 4.2. DNA Extraction, Library Preparation, and Sequencing

All isolates were cultured on Muller–Hinton agar (Bio-Rad Laboratories, Hercules, CA, USA) at 37 °C overnight prior to DNA extraction. Genomic DNA was extracted using the DNeasy Blood & Tissue Kit (Qiagen, Hilden, Germany). Sequencing was performed using a combination of Oxford Nanopore MinION (R10) and Illumina NextSeq platforms, as previously described [23]. The base-calling and demultiplexing of Nanopore reads were conducted using Dorado v5.0.16 [24] with the “sup” model.

### 4.3. Genome Assembly and Genotyping

Quality assessments of Illumina short reads and Nanopore long reads were performed using FastQC v0.11.9 [25] and MinIONQC v1.4.2 [26], respectively. Hybrid assemblies were generated with dragonflye v1.2.1 [27]. Assembly quality was assessed using QUAST v5.0.2 [28] and CheckM v1.2.4 [29]. Per-isolate values for raw Nanopore read coverage, raw Illumina read coverage, total assembly length, and N50 contig length are included in Supplementary Table S1. CheckM quality metrics (completeness and contamination) were also added; all genomes showed 100% completeness and 1% contamination. Taxonomic identification was carried out with Kraken2 v2.1.1 [30]. Genomes were further analyzed using the Type Strain Genome Server (TYGS, Leibniz Institute DSMZ, Braunschweig, Germany) [31]. A minimum contig size threshold of 500 bp was applied.

Genome annotation was performed with Prokka v1.14.5 [32]. Antimicrobial resistance genes were identified using AMRFinderPlus v4.0.23 [33]. Plasmid detection, typing, and clustering were conducted with MOB-suite [34]. Automated predictions were manually validated by confirming replicon markers, mo-bility genes, and contig structure. The oriTfinder tool v2.0 [35] was used to identify the origin of transfer (oriT) sites.

### 4.4. Comparative Genomics and Phylogenetic Analysis

*P. stuartii* genomes (*n* = 494) were downloaded from GenBank using the NCBI datasets command-line tool. The core genome alignment of the full dataset was performed using Parsnp v2.1.4 [36] (reference genome: 4016; recombination filtering with–x), and a maximum-likelihood tree was inferred with FastTree v2.1 (GTR model, 1000 bootstraps; midpoint-rooted) [37]. Population structure was inferred using FastBAPS [38]. Among the 494 genomes analyzed, the four study isolates were assigned to FastBAPS cluster 1 together with 39 publicly available genomes. Genomes from FastBAPS cluster 1 (the four study isolates and 39 additional publicly available genomes) were further analyzed with Panaroo v1.2.10 [39], which produces a filtered core genome alignment using the Block Mapping and Gathering with Entropy (BMGE) method. SNP positions were extracted using SNP-sites v2.5.1 [40], and a maximum-likelihood phylogeny was reconstructed using IQ-TREE v2.0.3 with ModelFinder [41,42] (TVM + e + ASC model; 1000 ultrafast bootstrap replicates; midpoint-rooted). Phylogenetic trees were visualized using Microreact [43] and iTOL v6 [44].

We used a custom plasmid database developed in our previous study, which combined all complete plasmids from GenBank with the complete plasmids sequenced in that study [21]. Pairwise comparisons with plasmids from this study were performed using FastANI v1.33 [45] to calculate average nucleotide identity (ANI) and alignment fraction (AF). Plasmids were considered highly similar if ANI > 99% and AF > 0.8. Both ANI/AF-based similarity metrics and their approximate k-mer-based analogs (e.g., Mash distances) are widely used for clustering plasmid sequences [46,47,48,49]. The stringent thresholds applied here (ANI ≥ 99%, AF ≥ 0.8) ensure that only near-identical plasmid backbones are grouped together.

### 4.5. Data Availability

All assemblies generated in this study were deposited in the NCBI database under BioProject accession PRJNA1321188.

## 5. Conclusions

This study provides a genomic characterization of NDM-producing *P. stuartii* from Russia and highlights the role of IncC plasmids in mediating carbapenem resistance within this species. The results underscore the importance of incorporating plasmid-level analyses into antimicrobial resistance monitoring frameworks, where cross-hospital or cross-regional transmission of plasmids may go undetected using strain-based approaches alone. Future research should include functional studies, such as conjugation assays, to evaluate plasmid transferability, in addition to expanded genomic surveillance across hospitals to track the dissemination of IncC backbones and other resistance-carrying plasmids over time. Strengthening genomic surveillance strategies that incorporate both chromosomal and plasmid analyses will be crucial for the early detection, containment, and prevention of emerging carbapenem-resistant *P. stuartii* and related threats in healthcare settings.

## Figures and Tables

**Table 1:** Case characteristics of NDM-producing *P. stuartii* cluster detected in June–July 2023.

ID	Age	Sex	Admit Date	Admitting Diagnosis	Source
4016	56	F	13 June 2023	Umbilical hernia with obstruction, without gangrene	Central venous catheter
4054	82	F	19 June 2023	Malignant neoplasm, unspecified site	Wound
4093	33	M	4 July 2023	Cutaneous abscess, furuncle and carbuncle of limb	Wound
4096	64	F	27 July 2023	Subarachnoid hemorrhage from middle cerebral artery	Wound

## Data Availability

All assemblies were deposited in the NCBI database under project accession number PRJNA1321188.

## Supplementary Materials

The following supporting information can be downloaded at https://www.mdpi.com/article/10.3390/antibiotics14121238/s1. Table S1: Phenotypic profile, sequencing, and assembly statistics. Table S2: Highly similar plasmids from the GenBank database. File S1: Figure S1: Core-genome phylogeny of *Providencia stuartii* genomes reconstructed with Parsnp. Figure S2: BRIG comparison of the representative IncC plasmid (4096_incC) with two K. pneumoniae plasmids.
