# Supplementary material for: Genomic Characterization of NDM-1 Producer Providencia stuartii Isolated in Russia
Source: Antibiotics (Basel). 2025 Dec 8;14(12):1238. doi: 10.3390/antibiotics14121238 (PMC12729570; doi:10.3390/antibiotics14121238)

Supplementary figure S1. Core-genome phylogeny of *Providencia stuartii* genomes. The core alignment was generated using Parsnp v2.1.4 with the chromosome of isolate 4016 used as the reference. Recombination filtering was enabled using the -x option. A maximum-likelihood phylogeny was reconstructed from the Parsnp alignment using FastTree v2.1 (GTR model, nucleotide mode) with 1,000 bootstrap replicates. The tree was midpoint-rooted, and bootstrap branch support values are shown at internal nodes. (A) Annotation of FastBAPS clusters. The FastBAPS cluster 1 clade, which includes the four outbreak isolates sequenced in this study, is denoted in red. (B) Additional overlaid annotations indicate (1) the blaNDM allele (categorical palette; white = absence), (2) the genomic context of blaNDM (chromosome or plasmid), and (3) MOB-typer primary cluster identifiers for plasmids predicted to harbor blaNDM.

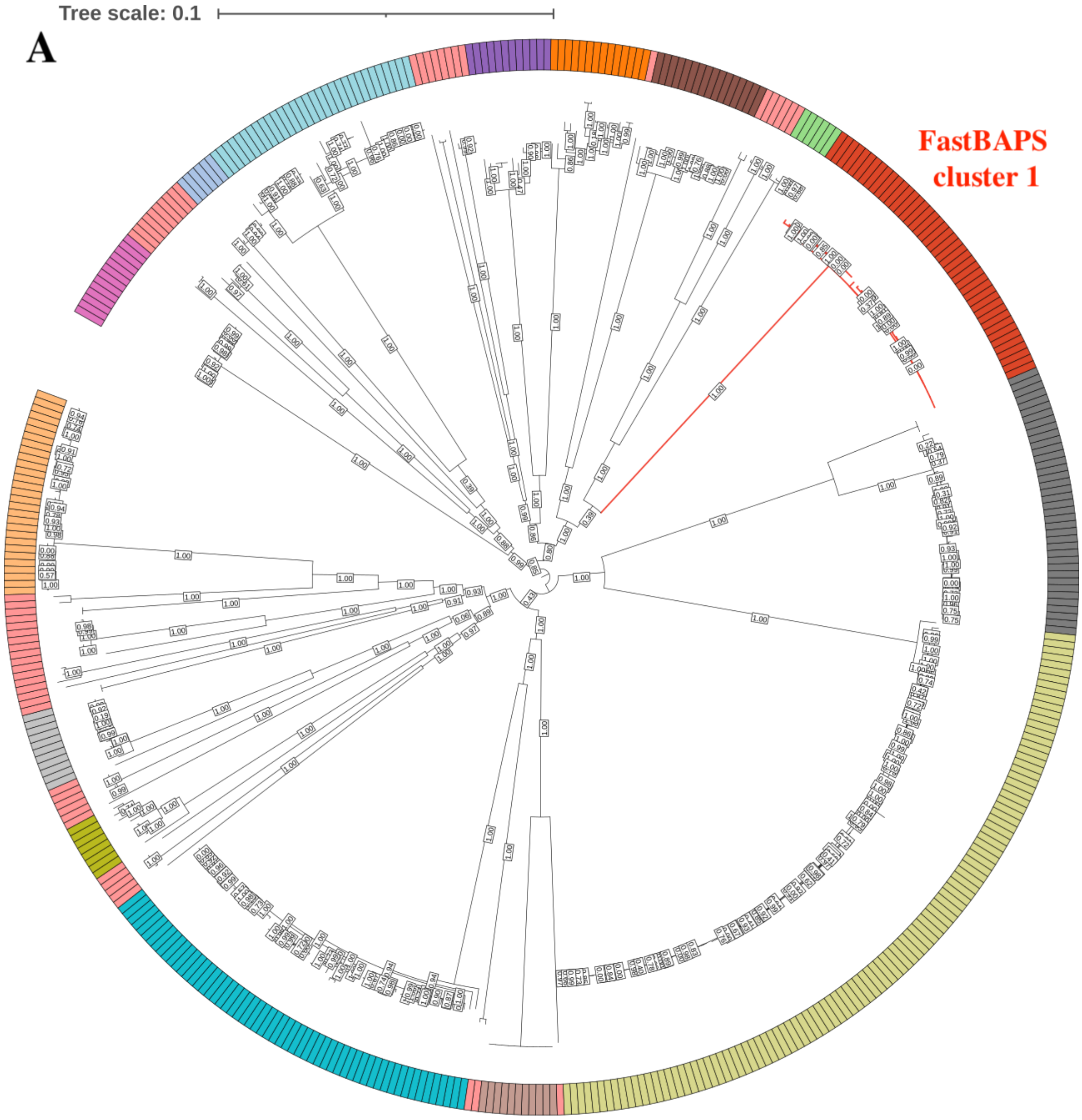

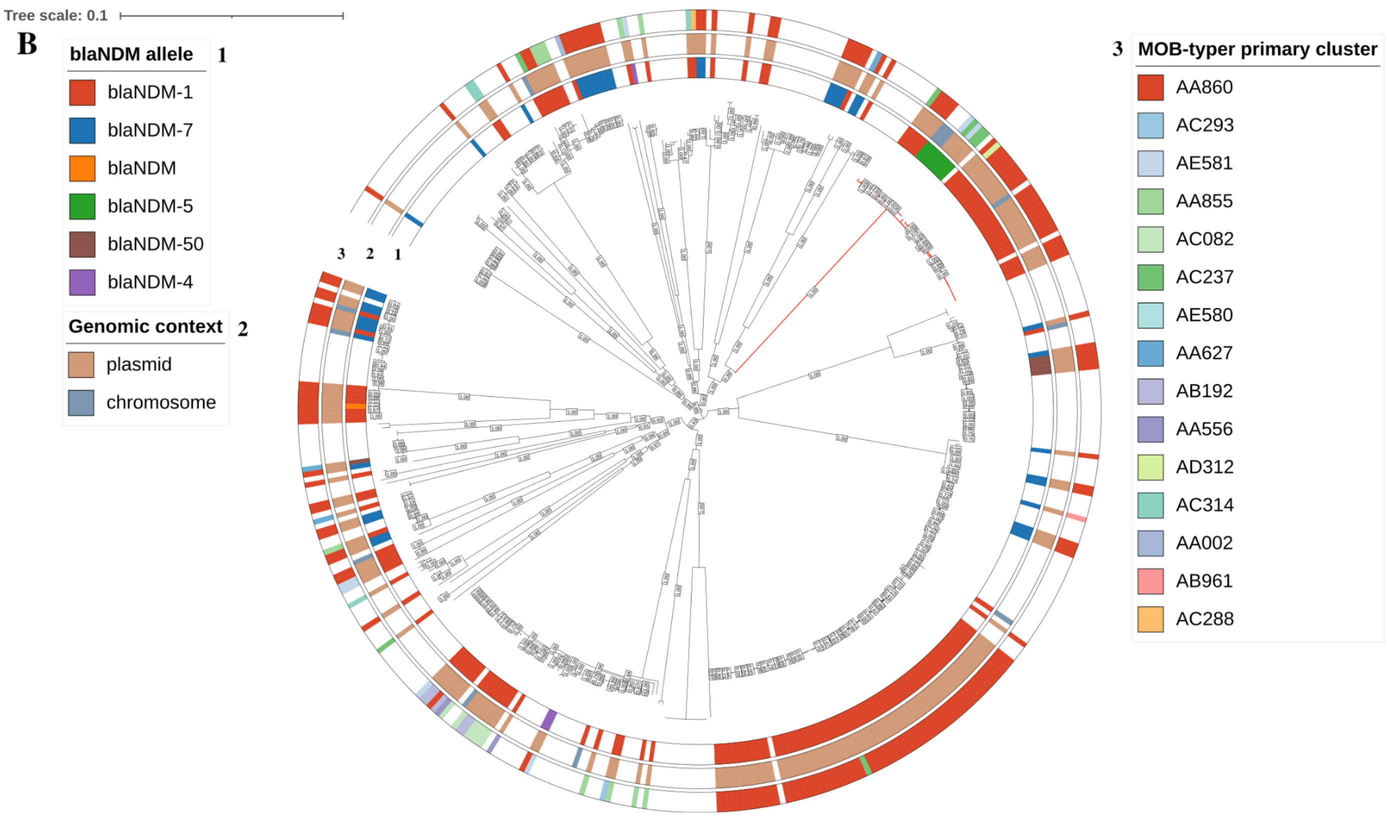

Supplementary Figure S2. BRIG comparison of the representative IncC plasmid (4096_incC) with two *K. pneumoniae* plasmids.

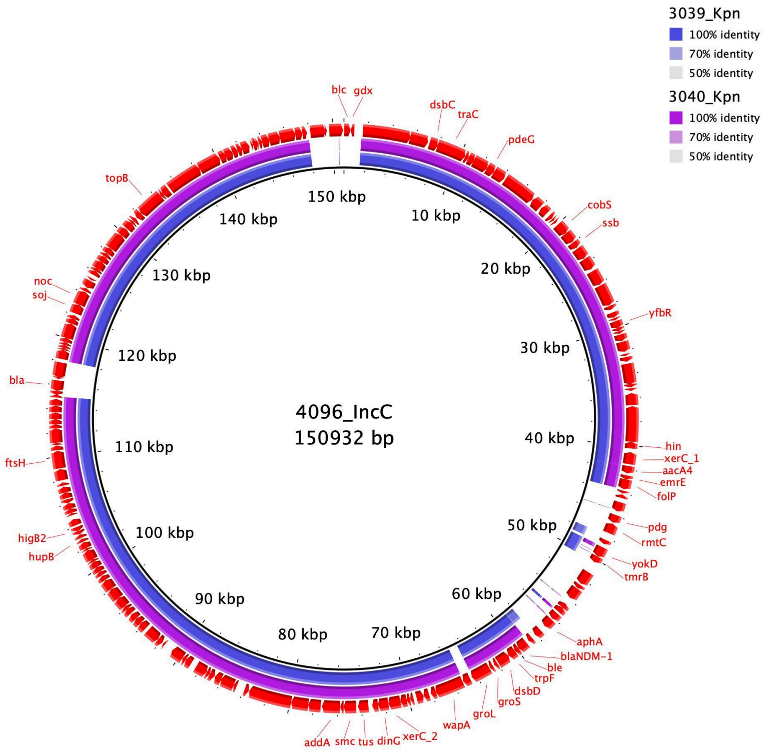

Supplement: Supplementary file 1 [file antibiotics-14-01238-s001.zip › supplementary_file_S1.pdf]
